# Osteosarcopenic obesity and its components—osteoporosis, sarcopenia, and obesity—are associated with blood cell count-derived inflammation indices in older Chinese people

**DOI:** 10.1186/s12877-022-03225-x

**Published:** 2022-06-28

**Authors:** Yi-zhen Nie, Zhao-qi Yan, Hui Yin, Ling-han Shan, Jia-hui Wang, Qun-hong Wu

**Affiliations:** 1 Physical Examination Center, The Second Affiliated Hospital of Harbin Medical University, Harbin, 150086 China; 2 Department of Health Education, School of Health Management, Harbin Medical University, Harbin, 150086 China; 3 School of Health Management, Harbin Medical University, Harbin, 150086 China; 4 Centre for Health Policy & Management, Health Management College, Harbin Medical University, 157 Baojian Road, Nangang District, Harbin, 150086 Heilongjiang Province People’s Republic of China

**Keywords:** Inflammation indices, Osteosarcopenic obesity, Osteoporosis, Sarcopenia, Obesity

## Abstract

**Background:**

The aim of this study was to investigate the associations of osteosarcopenic obesity (OSO) and its components with complete blood cell count-derived inflammation indices.

**Methods:**

In this cross-sectional study, data of 648 participants aged ≥60 years (men/women: 232/416, mean age: 67.21 ± 6.40 years) were collected from January 2018 to December 2020. Areal bone mineral density and body fat percentage were used to define osteopenia/osteoporosis and obesity, respectively. The criteria of the 2019 Asian Working Group for Sarcopenia were used to diagnose sarcopenia. Based on the number of these conditions, participants were divided into four groups: OSO/0, OSO/1, OSO/2, and OSO/3. Logistic regression analysis was conducted to identify associations between blood cell count-derived inflammation indices and the number of disorders with abnormal body composition.

**Results:**

Systemic inflammation response index (SIRI), white blood cells, neutrophil-to-lymphocyte ratio (NLR), aggregate inflammation systemic index (AISI), platelet-to-lymphocyte ratio (PLR), and lymphocyte-to-monocyte ratio (LMR) showed statistically significant differences among the four groups (*P <* 0.05). Unlike in the OSO/0 group, in all other groups, AISI, SIRI, PLR, and NLR were significantly associated with increased likelihood of having multiple disorders with abnormal body composition after adjustment for confounders (*P <* 0.0001 for all). However, LMR showed an inverse correlation with the number of these conditions (*P <* 0.05).

**Conclusion:**

Higher SIRI, AISI, NLR, and PLR values and lower LMR values are closely associated with OSO and its individual components—osteoporosis, sarcopenia, and obesity—in older adults, suggesting that the value of these indices in the evaluation of OSO warrants further investigation.

## Background

Osteoporosis, obesity, and sarcopenia are major global public health concerns [1]. A combination of two or three of these conditions usually results in comorbid conditions that pose an immense economic burden on older people and society [2, 3]. These three conditions have multifactorial etiologies and are believed to have some shared pathophysiological mechanisms involved in their development [3]. Given their close association with one another, a new clinical entity, osteosarcopenic obesity (OSO), has been proposed [4, 5]. As the age increases, body fat gets redistributed to the visceral organs and often infiltrates into muscle and bone tissues, potentially leading to sarcopenia and osteoporosis [6, 7]. Therefore, conventional evaluation tools often underestimate obesity in older adults [5]. Ethnicity also influences the distribution of body fat, particularly ectopic and visceral fat [8]. A study with a 5–10-year follow-up in community-dwelling adults demonstrated that sarcopenic obesity was associated with a higher risk for osteoporosis than obesity alone, indicating the presence of an interaction among the three conditions [9].

A hallmark of aging is a chronic state of low-grade systemic inflammation, also known as inflammaging, which has been shown to underlie the pathogenesis of various chronic disorders and comorbidities, including those comprising osteoporosis, obesity, and sarcopenia, ultimately contributing to frailty and disability [10, 11]. Inflammaging is usually accompanied by increased levels of proinflammatory mediators and their markers, such as interleukin-1β (IL-1β), tumor necrosis factor-α (TNF-α), C-reactive protein (CRP), and interleukin-6 (IL-6), reflecting destructive and degenerative processes in tissues [12]. As a major etiological factor for osteoporosis, estrogen deficiency favors the release of proinflammatory cytokines, particularly TNF-α, leading to preosteoclast production and bone resorption [13, 14]. In individuals with obesity, hypertrophied adipocytes secrete proinflammatory cytokines, which in turn promote the accumulation of proinflammatory immune cells [15]. Meanwhile, there is extracellular and intracellular lipid deposition, which replaces muscle fibers and damages myocyte contractile function [16]. Taken together, these mechanisms increase the risk of OSO [17].

Numerous peripheral blood biomarkers of inflammation, including IL-1β, TNF-α, CRP, and IL-6, have been used to monitor disease states and assess treatment outcomes [18–20]. Blood cell count-derived inflammation indices can be easily quantified using simple blood tests, which are a more economical and readily available alternative for the detection of chronic low-grade systemic inflammation and evaluation of chronic conditions. The cell types these indices are based on play critical roles in the host’s innate and adaptive immune responses to changes in the internal and external environments [21–25]. Some of the indices, such as the platelet-to-lymphocyte ratio (PLR), neutrophil-to-lymphocyte ratio (NLR), aggregate inflammation systemic index (AISI), and systemic inflammation response index (SIRI), have been used as inflammatory markers for characterizing many diseases, including osteoporosis, obesity, and sarcopenia [26–33]. Nevertheless, to our knowledge, their value in assessing OSO has not been investigated. In this study, we sought to establish the profiles of NLR, lymphocyte-to-monocyte ratio (LMR), PLR, AISI, and SIRI in patients with OSO and those with osteoporosis, obesity and/or sarcopenia.

## Methods

### Participants

This was a cross-sectional study involving 648 older adults (416 women, 232 men; age ≥ 60 years). Participants were randomly recruited from among people receiving annual check-ups at the Physical Examination Center of The Second Affiliated Hospital of Harbin Medical University between January 2018 and December 2020. This study was approved by the ethics committee of The Second Affiliated Hospital of Harbin Medical University (Ethics approval No: KY2018–262), and written informed consent was obtained from all participants. The exclusion criteria were as follows: (1) refusal to give or legally incapable of giving informed consent; (2) body composition and bone density could not be accurately estimated due to lower limb injuries; (3) history of serious metabolic or endocrine diseases or fractures; and (4) recent or ongoing use of medications affecting bone metabolism, such as thiazolidinediones, glucocorticoids, statins, and antiepileptic medications.

### Demographics, health-related behaviors, and health conditions

The following information was collected from in-person interviews: age, sex, education level (below university vs. university or higher), monthly household income (< ¥5000 vs. ≥¥5000), smoking, alcohol consumption, regular exercise, sunlight exposure (< 1 h/d vs. ≥1 h/d), and comorbidities (coronary heart disease, hypertension, stroke, diabetes, and fatty liver disease). A person was considered a smoker if the average number of cigarettes smoked per day in the past six months was ≥1. Alcohol consumption was defined as drinking at least once a week in the past six months [34]. Regular exercise was defined as exercise ≥5 times/week [35], with each exercise routine lasting at least 30 min.

### Physical and skeletal muscle measurements

An altimeter was used to measure the height of participants while they stood barefoot. The waist, calf, and hip circumferences of the participants were determined using a tape measure. For diastolic blood pressure and systolic blood pressure measurements, participants were asked to rest in a seated position for 5 min before their blood pressure values were acquired using an OMRON HEM-1000 electronic sphygmomanometer (OMRON Healthcare, Japan) by trained staff.

Dual-energy X-ray absorptiometry (Discovery-WI; Hologic Inc., Bedford-MA, USA) was used to estimate body composition. Data on total lean mass (kg), appendicular lean mass (kg), and total fat mass (in kg and %) were collected. Hand grip strength (HGS) was measured using a hand dynamometer (Jamar Hand Dynamometer, Sammons Preston, Inc., Bolingbrook, IL). Gait speed was calculated from a 4-m walk at normal pace and was expressed in meters/second (m/s). Areal bone mineral density (aBMD) was measured at the femur and the lumbar spine 1–4 (L1–L4).

### Laboratory tests

All laboratory tests were conducted at the Department of Laboratory Medicine, The Second Affiliated Hospital, Harbin Medical University. Fasting venous blood was drawn in the morning, and samples were processed on the same day. Standardized laboratory tests were performed for white blood cell counts, neutrophil counts, lymphocyte counts, monocyte counts, platelet counts, plasma glucose, albumin, plasma lipids, blood urea nitrogen (BUN), creatinine (Cr), and uric acid (UA).

### Diagnostic criteria for osteopenia, obesity, sarcopenia and their combinations

We defined sarcopenia as low skeletal muscle mass with low HGS or low gait speed (GS) or both on the basis of the recommended diagnostic algorithm of the 2019 consensus of the Asian Working Group for Sarcopenia (AWGS2019) [36]; we used AWGS2019 cutoff values to assess the participants. The appendicular skeletal mass index was calculated as the appendicular skeletal muscle mass/height^2^ (kg/m^2^). In line with the AWGS2019 recommendations, low skeletal muscle mass was evaluated using height-adjusted skeletal muscle mass from dual-energy X-ray absorptiometry (DXA), with cutoff values of 5.4 kg/m^2^ for women and 7.0 kg/m^2^ for men. AWGS2019 recommendations for low muscle strength are HGS values < 18.0 kg for women and < 28.0 kg for men [36]; a GS of < 1.0 m/s indicates reduced physical performance [36].

The WHO criteria were used for the diagnosis of osteopenia and osteoporosis. Osteopenia was defined as having an aBMD T-score between − 1.0 and − 2.5 SD and osteoporosis as having an aBMD T score of ≤ − 2.5 SD at the femoral neck, hip, and lumbar spine [5, 37]. The standard cutoff points recommended by the American Council on Exercise (ACE) were used to assess adiposity [38]. Obesity was defined as percent body fat (PBF) ≥32% for women and ≥ 25% for men [38].

Participants were divided into four groups on the basis of whether they had none, one, two, or all of the three conditions, namely obesity, osteoporosis, and sarcopenia: the OSO/0 group (none of the three conditions), the OSO/1 group (one condition), the OSO/2 group (two conditions), and the OSO/3 group (all three conditions).

### Calculation of inflammatory indices

AISI was calculated by multiplying the neutrophil count, monocyte count, and platelet count and dividing the result by the lymphocyte count [23]. SIRI was calculated by multiplying the neutrophil count and monocyte count and dividing the result by the lymphocyte count. LMR represented the ratio of the lymphocyte count over the absolute monocyte count. PLR is the ratio of the platelet count over the lymphocyte count, and NLR is the ratio of the neutrophil count over the lymphocyte count.

### Statistical analysis

All statistical analyses were performed using SAS 9.4 version (SAS Institute Inc., Cary, NC, USA). For baseline data, continuous variables were expressed as the mean ± SD or frequency (%), and categorical variables were expressed as an absolute number and percentage (%). We used the chi-square test (for categorical variables) or the Kruskal–Wallis test with the Bonferroni correction (for continuous variables) to examine differences between different groups. Where applicable, Spearman’s r or Pearson’s r was used to detect the correlations of inflammation indices with the parameters of OSO (GS, ASM, PBF, HGS, aBMD_femur_, and aBMD_L1-L4_). Logistic regression analysis was conducted to estimate the risk of osteoporosis, obesity, and/or sarcopenia based on blood cell-derived inflammation indices. First, univariate analysis was used to identify potential risk factors. Then, multivariate logistic regression analysis was performed to control for confounders. After adjustment for confounders, the odds ratios (ORs) and their 95% confidence intervals (CIs) for risk factors were calculated. All tests were two-tailed, with the statistical significance level set at *P <* 0.05.

## Results

The mean age of the 648 participants (64.2% women) was 67.21 ± 6.40 years; among these participants, 8.18% (53) belonged to the OSO/0 group, 30.25% (196) to the OSO/1 group, 45.06% (292) to the OSO/2 group, and 16.51% (107) to the OSO/3 group. Overall, 70.99% (460) had osteopenia or osteoporosis, 72.53% (470) had obesity, and 26.39% (171) had sarcopenia.

Osteoporosis, obesity, sarcopenia, or any combination of these disorders was positively correlated with age (*P <* 0.001), coronary heart disease (*P* = 0.001), PBF (*P <* 0.001), and total cholesterol (TCH, P = 0.001) and low-density lipoprotein (LDL, *P* = 0.019) levels but negatively correlated with alcohol consumption, regular exercise, duration of sunlight exposure, creatinine, aBMD_L1-L4_, aBMD_total hip_, aBMD_femoral neck_, appendicular skeletal mass index (ASMI), HGS, and GS (*P <* 0.001 for all; Table 1).

**Table 1:** General characteristics of participants with different numbers of osteoporosis, obesity, and sarcopenia

	OSO/0	OSO/1	OSO/2	OSO/3	*P-*value
Age (years)	64 (61–66)	65 (61–69)	66 (62–72)*	69 (63–76)*†‡	**< 0.001**
Sex					**< 0.001**
Men	40 (75.47)	98 (50)	67 (22.95)	28 (26.17)	
Women	13 (24.53)	98 (50)*	225 (77.05)*†	79 (73.83)*†	
Education level					0.245
< College	26 (49.06)	109 (55.61)	181 (61.99)	64 (59.81)	
≥ College	27 (50.94)	87 (44.39)	111 (38.01)	43 (40.19)	
Monthly household income					**0.026**
< ¥5000	32 (60.38)	120 (61.22)	213 (72.95)	76 (71.03)	
≥ ¥5000	21 (39.62)	76 (38.78)	79 (27.05)†	31 (28.97)†	
smoking n (%)					0.230
No	40 (75.47)	160 (81.63)	248 (84.93)	93 (86.92)	
Yes	13 (24.53)	36 (18.37)	44 (15.07)	14 (13.08)	
Alcohol consumption n (%)					**< 0.001**
No	26 (49.06)	138 (70.41)	250 (85.62)	101 (94.39)	
Yes	27 (50.94)	58 (29.59)*	42 (14.38)*†	6 (5.61)*†	
Hypertension n (%)					0.630
No	31 (58.49)	98 (50.00)	160 (54.79)	56 (52.34)	
Yes	22 (41.51)	98 (50.00)	132 (45.21)	51 (47.66)	
Coronary heart disease n (%)					**0.001**
No	42 (79.25)	149 (76.02)	201 (68.84)	60 (56.07)	
Yes	11 (20.75)	47 (23.98)	91 (31.16)	47 (43.93)*	
Diabetes n (%)					**0.004**
No	44 (83.02)	145 (73.98)	245 (83.90)	96 (89.72)	
Yes	9 (16.98)	51 (26.02)	47 (16.10)†	11 (10.28)†	
Stroke n (%)					0.484
No	28 (52.83)	90 (45.92)	144 (49.32)	45 (42.06)	
Yes	25 (47.17)	106 (54.08)	148 (50.68)	62 (57.94)	
Fatty liver disease n (%)					**0.006**
No	21 (39.62)	51 (26.02)	61 (20.89)	36 (33.64)	
Yes	32 (60.38)	145 (73.98)	231 (79.11)*	71 (66.36)	
Regular exercise n (%)					**< 0.001**
No	13 (24.53)	88 (44.90)	162 (55.48)	76 (71.03)	
Yes	40 (75.47)	108 (55.10)*	130 (44.52)*	31 (28.97)*‡	
Sunlight exposure					**< 0.001**
< 1 h/d	5 (9.43)	48 (24.49)	102 (34.93)	65 (60.75)	
≥ 1 h/d	48 (90.57)	148 (75.51)	190 (65.07)*	42 (39.25)*‡	
aBMD_L1-L4_ (g/cm^2^)	1.07 (1.03–1.16)	0.99 (0.86–1.10)*	0.83 (0.75–0.92)*†	0.78 (0.70–0.87)*†‡	**< 0.001**
aBMD_total hip_ (g/cm^2^)	1.03 (0.97–1.07)	0.91 (0.82–0.99)*	0.78 (0.69–0.86)*†	0.73 (0.67–0.82)*†‡	**< 0.001**
aBMD_femoral neck_ (g/cm^2^)	0.87 (0.81–0.92)	0.77 (0.67–0.84)*	0.65 (0.58–0.73)*†	0.60 (0.55–0.67)*†‡	**< 0.001**
ASMI (kg/m^2^)	7.60 (7.11–8.22)	6.91 (6.15–7.61)*	6.08 (5.67–6.71)*†	5.20 (5.05–5.39)*†‡	**< 0.001**
Handgrip strength (kg)	31.80 (24.40–35.30)	23.50 (17.95–31.40)*	18.60 (15.55–22.45)*†	16.50 (14.30–18.00)*†‡	**< 0.001**
Gait speed (m/s)	1.33 (1.23–1.43)	1.23 (1.14–1.32)*	1.16 (1.02–1.25)*†	0.79 (0.75–0.89)*†‡	**< 0.001**
Body fat (%)	23.50 (21.20–24.60)	29.00 (24.90–34.25)*	35.00 (31.80–38.35)*†	36.00 (32.30–37.90)*†	**< 0.001**
Laboratory data					
TG	1.24 (0.99–1.62)	1.59 (1.12–2.30)	1.53 (1.11–2.21)	1.60 (1.12–2.10)	**0.048**
TCH	4.51 (4.25–5.47)	4.90 (4.29–5.61)	5.24 (4.50–5.90)	5.25 (4.65–5.91)	**0.001**
LDL	2.64 (2.25–3.13)	2.81 (2.24–3.32)	2.97 (2.38–3.56)	3.03 (2.47–3.54)	**0.019**
HDL	1.27 (1.17–1.50)	1.23 (1.03–1.47)	1.31 (1.13–1.58)	1.31 (1.13–1.58)	**0.031**
Albumin	46.30 (45.10–47.60)	45.80 (44.00–47.60)	45.60 (44.10–47.30)	45.80 (44.10–47.40)	0.200
UA	335.00 (272.00–374.00)	327.50 (276.00–379.00)	290.00 (246.50–347.50)†	293.00 (253.00–336.00)†	**< 0.001**
Creatinine	82.00 (74.00–89.00)	75.00 (63.50–83.00)	66.00 (57.00–76.50)*†	65.00 (56.00–75.00)*†	**< 0.001**
BUN	5.42 (4.56–6.58)	5.63 (4.75–6.67)	5.45 (4.68–6.40)	5.38 (4.58–6.23)	0.383

Categorical variables were expressed as an absolute number and percentage (%) of the total; continuous variables were expressed as frequency (percentage). Differences between groups were examined using the chi-square test (for categorical variables) or the Kruskal–Wallis test with the Bonferroni correction (for continuous variables)

*Abbreviations*: *d* Day, *TG* Triglycerides, *TCH* Total cholesterol, *LDL* Low-density lipoprotein cholesterol, *HDL* High-density lipoprotein cholesterol, *UA* Uric acid, *BUN* Blood urea nitrogen, *aBMD* Bone mineral density, *aBMD*_*L1-L4*_ Bone mineral density at the lumbar spine 1–4

*P < 0.05 compared with the normal group

†*P <* 0.05 compared with the group with one disorder

‡*P <* 0.05 compared with the group with two disorders

The relationships of inflammatory indices with osteoporosis, obesity, sarcopenia, and their combinations are shown in Table 2. The Kruskal–Wallis test with the Bonferroni correction revealed statistically significant differences in white blood cells (WBC), SIRI, AISI, NLR, PLR, and LMR among the four groups (*P <* 0.05). Higher numbers of these disorders were associated with higher AISI, SIRI, and NLR values and lower LMR values (*P <* 0.001 for each index). Paired comparisons between groups showed that the OSO/3 group had higher AISI, SIRI, and NLR values than any other group (*P <* 0.05). WBC counts were lower in the OSO/0 group than in any other group. Furthermore, SIRI, AISI, NLR, and PLR values were higher and LMR values were lower in the OSO/1, OSO/2, and OSO/3 groups than in the OSO/0 group (*P <* 0.05 for each index).

**Table 2:** Relationships between inflammatory indices and numbers of disorders with body composition

	OSO/0	OSO/1	OSO/2	OSO/3	*P-*value
WBC	5.60 (4.70–7.00)	6.20 (5.40–7.30)	6.20 (5.20–7.20)	6.90 (5.60–7.90)*	0.005
SIRI	0.38 (0.27–0.54)	0.57 (0.43–0.82)*	0.59 (0.43–0.78)*†	0.75 (0.56–0.98)*†‡	< 0.001
AISI	77.76 (51.10–125.57)	129.68 (102.77–198.49)*	142.64 (98.60–192.39)*	188.60 (132.53–236.71)*†‡	< 0.001
NLR	1.42 (1.21–1.60)	1.91 (1.72–2.23) *	1.95 (1.75–2.22) *	2.20 (1.99–2.61) *†‡	< 0.001
PLR	97.56 (80.63–120.36)	123.30 (103.57–152.69) *	131.69 (109.75–158.71) *	127.96 (109.35–161.19) *	< 0.001
LMR	7.44 (5.86–10.42)	6.07 (4.65–7.81)*	6.15 (4.88–7.98)*	5.33 (4.37–6.83)*‡	< 0.001

Continuous variables were expressed as frequency (percentage). The Kruskal–Wallis test with the Bonferroni correction was used to examine differences between groups

*Abbreviations*: *PLR* Platelet-to-lymphocyte ratio, *NLR* Neutrophil-to-lymphocyte ratio, *WBC* White blood cells, *LMR* Lymphocyte-to-monocyte ratio, *AISI* Aggregate index of systemic inflammation, *SIRI* Systemic inflammation response index

*P < 0.05 compared with the normal group

†*P <* 0.05 compared with the group with one disorder

‡*P >* 0.05 compared with the group with two disorders

Correlations of the inflammatory indices with ASMI, HGS, GS, PBF, aBMD_L1-L4_, aBMD_total hip_ and aBMD_femoral neck_ were identified using Spearman’s and Pearson’s analyses (Table 3). Overall, no clear correlation patterns were identified between most of the inflammatory indices and parameters used for the assessment of osteoporosis, obesity, and sarcopenia in different groups. The WBC count was not correlated with any of the parameters in any of the groups. SIRI, AISI, and PLR were correlated with some of the parameters, but the correlations did not become stronger with increasing numbers of disorders. NLR was correlated with aBMD_total hip_ (*r* = 0.153, *P* = 0.032) and aBMD_femoral neck_ (*r* = 0.167, *P* = 0.019) in the OSO/1group, whereas LMR was correlated with ASMI (*r* = − 0.250, *P* = 0.009) and HGS (*r* = − 0.202, *P* = 0.037) in the OSO/3 group.

**Table 3:** Correlations of inflammation indices with common parameters for osteoporosis, obesity, and sarcopenia assessment

		ASMI r P	HGS r P	GS r P	PBF r P	aBMD_L1-L4_, r P	aBMD_total hip_ r P	aBMD_femoral neck_ r P
0	WBC	0.373	0.191	0.209	− 0.158	0.027	− 0.055	0.034
		0.006	0.172	0.132	0.259	0.851	0.697	0.809
1		0.130	0.060	−0.068	− 0.080	0.030	− 0.037	0.014
		0.068	0.400	0.341	0.265	0.678	0.604	0.840
2		0.097	0.029	−0.062	−0.065	− 0.001	− 0.048	− 0.066
		0.097	0.626	0.295	0.270	0.983	0.414	0.262
3		−0.057	−0.059	0.104	0.095	−0.039	0.021	0.014
		0.562	0.545	0.287	0.333	0.691	0.833	0.882
0	SIRI	0.142	0.076	0.196	0.080	0.090	−0.080	0.089
		0.309	0.588	0.160	0.567	0.522	0.568	0.528
1		0.238	0.163	−0.112	−0.225	0.060	0.020	0.094
		**0.001**	**0.022**	0.117	**0.002**	0.407	0.778	0.188
2		0.155	0.078	−0.153	−0.200	0.047	0.100	0.079
		**0.008**	0.186	**0.009**	**0.001**	0.423	0.088	0.177
3		0.158	0.199	0.040	−0.013	0.015	0.083	0.069
		0.105	**0.040**	0.683	0.893	0.882	0.395	0.482
0	AISI	0.173	−0.017	0.150	0.192	0.044	−0.136	0.058
		0.216	0.905	0.285	0.169	0.753	0.333	0.679
1		0.163	0.082	−0.131	−0.133	0.003	0.004	0.083
		**0.022**	0.251	0.066	0.063	0.965	0.960	0.250
2		0.084	0.029	−0.131	−0.135	−0.035	0.060	0.062
		0.153	0.617	**0.025**	**0.021**	0.552	0.309	0.294
3		0.042	0.081	0.014	0.121	−0.052	0.075	0.027
		0.668	0.408	0.889	0.214	0.595	0.440	0.785
0	NLR	−0.118	−0.035	0.130	0.160	−0.025	0.050	0.150
		0.399	0.802	0.353	0.251	0.862	0.724	0.454
1		0.085	−0.002	−0.095	−0.103	0.110	0.153	0.167
		0.238	0.972	0.185	0.152	0.125	**0.032**	**0.019**
2		−0.007	−0.043	− 0.219	−0.106	− 0.039	−0.033	− 0.099
		0.905	0.464	<0.001	0.071	0.505	0.569	0.092
3		0.032	0.217	0.050	−0.023	0.028	0.144	0.142
		0.745	**0.025**	0.607	0.812	0.777	0.139	0.144
0	PLR	−0.181	−0.173	−0.069	0.227	0.042	−0.044	0.031
		0.195	0.216	0.623	0.101	0.768	0.754	0.827
1		−0.136	−0.175	−0.097	0.106	−0.067	0.038	0.062
		0.057	**0.014**	0.175	0.139	0.353	0.599	0.392
2		−0.193	−0.125	−0.014	0.078	−0.097	− 0.0297	−0.010
		**0.001**	**0.033**	0.807	0.184	0.099	0.614	0.860
3		−0.027	0.049	−0.019	0.097	−0.047	0.049	0.001
		0.786	0.618	0.848	0.319	0.633	0.616	0.990
0	LMR	−0.088	−0.054	−0.093	− 0.150	−0.122	0.098	−0.104
		0.532	0.701	0.508	0.284	0.384	0.485	0.459
1		−0.149	−0.132	0.111	0.126	−0.059	−0.022	− 0.099
		**0.038**	0.066	0.120	0.078	0.411	0.76	0.169
2		−0.114	−0.049	0.094	0.164	−0.055	−0.105	− 0.114
		0.052	0.401	0.110	**0.005**	0.347	0.074	0.051
3		−0.250	−0.202	0.135	0.035	−0.042	−0.133	− 0.105
		**0.009**	**0.037**	0.165	0.723	0.667	0.172	0.281

*Abbreviations*: *ASMI* Appendicular skeletal muscle mass index, *WBC* White blood cells, *SIRI* Systemic inflammation response index, *AISI* Aggregate index of systemic inflammation, *NLR* Neutrophil-to-lymphocyte ratio, *PLR* Platelet-to-lymphocyte ratio, and *LMR* Lymphocyte-to-monocyte ratio, *ASMI* Appendicular lean mass index, *HGS* Handgrip strength, *GS* Gait speed, *aBMD*_*L1–4*_, Bone mineral density at the lumbar spine1 to 4, *aBMD*_*total hip*_, Bone mineral density at the total hip, *aBMD*_*femoral neck*_ Bone mineral density at femoral neck

Spearman’s r was used for correlations between non-normal distribution variables and Pearson’s r for correlations between normal distribution variables, and *P <* 0.05 was considered statistically significant

Using data in the OSO/0 group as normal reference values, logistic regression analyses were performed to evaluate the potential of the inflammatory indices as indicators of the risk of osteoporosis, obesity, sarcopenia, or their combinations. After adjustment for confounding factors, SIRI, AISI, NLR, and PLR were found to be positively correlated with the presence of one or more of the following conditions: osteoporosis, obesity, and sarcopenia (*P <* 0.001 for OSO/1, OSO/2 and OSO/3); however, LMR was negatively correlated with OSO/1, OSO/2, and OSO/3 (*P <* 0.05). In addition, WBC was associated with OSO/3 (OR = 1.410, 95% CI: 1.111–1.789; Table 4).

**Table 4:** Multivariate logistic regression analysis on the predictive value of inflammation indices for osteoporosis, obesity, sarcopenia assessment and their combinations

Variable	Crude OR (95% CI)	*P-*value	Adjusted OR (95% CI)	*P-*value
WBC				
OSO/1	1.176 (0.969–1.426)	0.100	1.174 (0.947*–*1.456)	0.144
OSO/2	1.191 (0.988*–*1.437)	0.067	1.242 (0.998*–*1.546)	0.052
OSO/3	1.342 (1.098*–*1.640)	0.004	1.410 (1.111*–*1.789)	0.005
SIRI				
OSO/1	93.919 (18.734*–*470.854)	< 0.001	90.225 (15.146*–*537.469)	< 0.001
OSO/2	56.043 (11.376*–*276.083)	< 0.001	76.861 (12.668*–*466.326)	< 0.001
OSO/3	167.699 (32.781*–*857.895)	< 0.001	247.651 (39.090*–*1568.985)	< 0.001
AISI				
OSO/1	1.020 (1.013*–*1.026)	< 0.001	1.018 (1.011*–*1.026)	< 0.001
OSO/2	1.018 (1.012*–*1.025)	< 0.001	1.018 (1.011*–*1.025)	< 0.001
OSO/3	1.022 (1.015*–*1.028)	< 0.001	1.021 (1.014*–*1.029)	< 0.001
NLR				
OSO/1	25.191 (10.666*–*59.496)	< 0.001	61.909 (18.317*–*209.245)	< 0.001
OSO/2	25.871 (11.084*–*60.385)	< 0.001	72.926 (21.418–248.309)	< 0.001
OSO/3	54.583 (22.596*–*131.848)	< 0.001	165.623 (46.879*–*585.150)	< 0.001
PLR				
OSO/1	1.025 (1.014*–*1.035)	< 0.001	1.026 (1.014*–*1.039)	< 0.001
OSO/2	1.030 (1.019*–*1.040)	< 0.001	1.029 (1.016*–*1.042)	< 0.001
OSO/3	1.031 (1.020*–*1.042)	< 0.001	1.030 (1.016*–*1.044)	< 0.001
LMR				
OSO/1	0.861 (0.787*–*0.942)	0.001	0.845 (0.771*–*0.927)	< 0.001
OSO/2	0.895 (0.828*–*0.969)	0.006	0.876 (0.806*–*0.952)	0.002
OSO/3	0.737 (0.652*–*0.834)	< 0.001	0.734 (0.638*–*0.845)	< 0.001

Data from the OSO/0 group were used as normal reference values and logistic regression analyses were conducted to evaluate the potential of the inflammatory indices as surrogates for the risk of disorders with abnormal body composition, with *P <* 0.05 considered statistically significant

Adjustment was made for sex, monthly household income, regular exercise, age, diabetes, coronary heart disease, alcohol consumption, sunlight exposure, creatinine, fatty liver disease, TG, UA, TCH, LDL, and HDL

## Discussion

This study was intended to assess the value of several common inflammatory markers, namely, SIRI, WBC, NLR, AISI, LMR, and PLR, in screening patients with osteoporosis, obesity, sarcopenia, or their combinations. Lower LMR values and higher AISI, SIRI, and NLR values were associated with a higher number of these disorders. Participants belonging to the OSO/3 group had higher AISI, SIRI, and NLR values than those belonging to the OSO/1 or OSO/2 group. After adjustment for confounding factors, these inflammatory markers, except for WBC, were independently correlated with these disorders. Furthermore, AISI, SIRI, PLR, NLR, and LMR were moderately associated with HGS, ASMI, PBF, and GS, which are often used to evaluate sarcopenia and body composition. Our study was the first to use these markers to characterize comorbidities comprising osteoporosis, obesity, and sarcopenia, and we generated promising leads for further validation of these markers in the assessment of the above disorders.

Osteoporosis, obesity, and sarcopenia have been studied extensively as individual conditions, and strong evidence has been uncovered for the roles of inflammatory cytokines, such as IL-6, IL-1, and TNF-α, in their pathogenesis [39–41]. Osteoporosis represents an imbalance in bone metabolism wherein bone absorption exceeds bone formation [42]. Its development requires the participation of inflammatory cytokines, which promote the formation of osteoclasts from its precursors through a series of signal transduction pathways [43–46]. Higher circulating levels of proinflammatory cytokines have been found to be associated with a lower aBMD, accelerated bone loss, and increased fracture risk in older adults [47–49]. Obesity is not only a major feature of inflammation but also a contributor to a chronic inflammatory state [50]. Increased levels of TNF-α, IL-6, IL-1β, and other cytokines are found in adipose tissues of obese animals and humans [51]. The mechanisms responsible for sarcopenia are poorly understood because of lack of appropriate animal models [52]. However, loss of muscle mass, muscle strength, and physical performance are generally accompanied by increased circulating levels of proinflammatory cytokines [53]. These markers are often not included in the diagnostic criteria for these disorders.

Another reason behind choosing these inflammatory markers in the present study was that although they have been increasingly used for evaluating chronic inflammatory diseases and monitoring cancer therapy, their value in osteoporosis, obesity, and sarcopenia remains to be firmly established because of the limited number of studies [54, 55]. NLR has been reported to be negatively associated with aBMD; however, this is not reflected in our results [56]. It has also been suggested as an indicator of the risk of postmenopausal osteopenia in women [57]. Since obesity-related inflammation with adipose tissue remodeling occurs predominantly in individuals with visceral adiposity, which causes monocyte/macrophage infiltration and changes in lymphocyte subtypes, biomarkers based on these cell types may very well reflect these dynamic processes [32, 58]. Indeed, a study of metabolic syndrome in middle-aged and older adults in Taiwan showed that PBF, obesity, hip circumference, waist circumference, and waist-to-hip ratio had a significant positive correlation with NLR and C-reactive protein levels [59]. Sarcopenia and systemic inflammatory indices have been used together as indicators for outcomes of cancer treatment [60]. The potential of these indices as tools for the assessment of sarcopenia remains unclear.

Our results showed that some of the demographic parameters (age, sex, and monthly household income), health-related habits (alcohol consumption, regular exercise, and sunlight exposure), laboratory test results (TG, TCH, LDL, HDL, UA, and Cr levels), and medical conditions (coronary heart disease, diabetes, and fatty liver) were associated with the prevalence of osteoporosis, obesity, sarcopenia, and their combinations. As shown in previous studies, these disorders are more likely to occur in aging individuals with unhealthy habits, metabolic abnormalities, or chronic diseases [2, 61]. It is particularly interesting that Cr and UA levels were lower in groups with abnormal body composition. Cr is often used to estimate renal function, but as a metabolite from skeletal muscle, its levels are dependent on muscle mass under steady renal function [62]. However, Cr levels may also be affected by sex and age [63]. Women and older adults typically have relatively low muscle mass and therefore a low output of Cr [64, 65]. As we had large proportions of women and older adults in the study and sarcopenia is more likely to be found in people with a multiple disorders with abnormal body composition, the findings of Cr largely reflect these characteristics. UA is the end-product of purine metabolism, and its effects on muscle function are far from unequivocal. Serum UA levels are positively associated with levels of proinflammatory cytokines, such as TNF-α and IL-6 [66]. Conversely, UA is a powerful scavenger of reactive oxygen species, which possess proinflammatory properties [67]. In our study, UA levels were lower in the OSO/2 and OSO/3 groups than in the OSO/1 group, suggesting a positive correlation with aBMD and muscle mass [68–71].

This study had some limitations. First, we only collected cross-sectional data, and therefore, we were unable to establish any causal relationships between blood cell count-derived inflammation indices and OSO. Second, as a single-center study with a moderate sample size, some potential biases may not have been completely eliminated. Third, there were nearly twice as many women as men in the study, and the sex distribution was clearly not representative of that of the general population. Additionally, as there were no standard PBF cutoffs available for the Chinese population, we used values recommended by the ACE, which may have underestimated obesity due to ethnic differences. Finally, this study explored the association of OSO and its components with blood cell count-derived inflammation indices in older Chinese adults. Considering that lifestyle is an important etiological factor for these disorders, our findings need confirmation from studies on populations in other countries. The value of these inflammation indices as assessment tools and indicators of treatment efficacy for OSO will become clearer when larger-scale and prospective data are available.

## Conclusions

After accounting for potential confounding factors, we found the blood cell count-derived inflammation indices SIRI, NLR, PLR, AISI, and LMR to be associated with increased prevalence of OSO. These findings suggest that the value of these indices in the evaluation of these disorders warrants further investigation.

## Data Availability

The datasets generated and analyzed during the present study are available from the corresponding author on reasonable request.

## Abbreviations

ACE: The American Council on Exercise
AISI: Aggregate inflammation systemic index
ASMI: Appendicular skeletal mass index
AWGS: The Asian Working Group for Sarcopenia
BUN: Blood urea nitrogen
aBMD: Bone mineral density
CI: Confidence interval
Cr: Creatinine
HDL: High-density lipoprotein cholesterol
HGS: Handgrip strength
GS: Gait speed
LDL: Low-density lipoprotein cholesterol
LMR: Lymphocyte-to-monocyte ratio
NLR: Neutrophil-to-lymphocyte ratio
OR: Odds ratios
OSO: Osteosarcopenic obesity
PBF: Percentage body fat
PLR: Platelet-to-lymphocyte ratio
SIRI: Systemic inflammation response index
TCH: Total cholesterol
TG: Triglycerides
UA: Uric acid
WBC: White blood cell
